# Evaluation of a novel deep learning–based classifier for perifissural nodules

**DOI:** 10.1007/s00330-020-07509-x

**Published:** 2020-12-02

**Authors:** Daiwei Han, Marjolein Heuvelmans, Mieneke Rook, Monique Dorrius, Luutsen van Houten, Noah Waterfield Price, Lyndsey C. Pickup, Petr Novotny, Matthijs Oudkerk, Jerome Declerck, Fergus Gleeson, Peter van Ooijen, Rozemarijn Vliegenthart

**Affiliations:** 1 University Medical Center Groningen, Department of Radiology, University of Groningen, Groningen, The Netherlands; 2 University Medical Center Groningen, Department of Epidemiology, University of Groningen, Groningen, The Netherlands; 3 Department of Pulmonology, Medisch Spectrum Twente, Enschede, The Netherlands; 4 Department of Radiology, Martini Ziekenhuis, Groningen, The Netherlands; 5 Optellum Ltd, Oxford, UK; 6 Faculty of Medical Sciences, University of Groningen, Groningen, the Netherlands; 7 Institute for Diagnostic Accuracy, Groningen, The Netherlands; 8 National Consortium of Intelligent Medical Imaging, Oxford University, Oxford, Great Britain UK; 9 University Medical Center Groningen, Department of Radiotherapy, University of Groningen, Groningen, The Netherlands

**Keywords:** Tomography, X-ray computed, Solitary pulmonary nodule, Deep learning

## Abstract

**Objectives:**

To evaluate the performance of a novel convolutional neural network (CNN) for the classification of typical perifissural nodules (PFN).

**Methods:**

Chest CT data from two centers in the UK and The Netherlands (1668 unique nodules, 1260 individuals) were collected. Pulmonary nodules were classified into subtypes, including “typical PFNs” on-site, and were reviewed by a central clinician. The dataset was divided into a training/cross-validation set of 1557 nodules (1103 individuals) and a test set of 196 nodules (158 individuals). For the test set, three radiologically trained readers classified the nodules into three nodule categories: typical PFN, atypical PFN, and non-PFN. The consensus of the three readers was used as reference to evaluate the performance of the PFN-CNN. Typical PFNs were considered as positive results, and atypical PFNs and non-PFNs were grouped as negative results. PFN-CNN performance was evaluated using the ROC curve, confusion matrix, and Cohen’s kappa.

**Results:**

Internal validation yielded a mean AUC of 91.9% (95% CI 90.6–92.9) with 78.7% sensitivity and 90.4% specificity. For the test set, the reader consensus rated 45/196 (23%) of nodules as typical PFN. The classifier-reader agreement (*k* = 0.62–0.75) was similar to the inter-reader agreement (*k* = 0.64–0.79). Area under the ROC curve was 95.8% (95% CI 93.3–98.4), with a sensitivity of 95.6% (95% CI 84.9–99.5), and specificity of 88.1% (95% CI 81.8–92.8).

**Conclusion:**

The PFN-CNN showed excellent performance in classifying typical PFNs. Its agreement with radiologically trained readers is within the range of inter-reader agreement. Thus, the CNN-based system has potential in clinical and screening settings to rule out perifissural nodules and increase reader efficiency.

**Key Points:**

*• Agreement between the PFN-CNN and radiologically trained readers is within the range of inter-reader agreement.*

*• The CNN model for the classification of typical PFNs achieved an AUC of 95.8% (95% CI 93.3–98.4) with 95.6% (95% CI 84.9–99.5) sensitivity and 88.1% (95% CI 81.8–92.8) specificity compared to the consensus of three readers.*

**Supplementary Information:**

The online version contains supplementary material available at 10.1007/s00330-020-07509-x.

## Introduction

Since the publication of the National Lung Screening Trial (NLST) demonstrated a reduction in lung cancer–related mortality of 20% compared to chest X-ray [1], and this has been confirmed more recently by European studies [2, 3], resulting in a significant amount of interest on lung cancer screening using computed tomography. Alongside the detection of lung cancer, the increased use of low-dose computed tomography (CT) for screening purposes has resulted in the increased detection of benign of small-to-intermediate-sized pulmonary in approximately 50% of the high-risk individuals screened [4], resulting in a substantial number of false-positive results [5].

Perifissural nodules (PFN) are a sub-group of small-to-intermediate-sized solid nodules, frequently identified by radiologists reporting chest CT scans. PFNs are defined as nodules that are attached to fissures, and are homogenous and solid with smooth margins and an oval/lentiform or triangular shape [6]. They account for approximately 20–30% of all solid pulmonary nodules found in the lung cancer screening setting as well as incidentally in the clinical setting [6–8]. Although some of the PFNs show growth rates similar to malignant nodules (volume doubling time < 400 days), they have so far been shown to be benign in all typical cases [6–9], and are generally considered to be benign reactive intrapulmonary lymph nodes [10, 11]. Automated and consistent identification of PFNs with their exclusion from further consideration could reduce the workload of radiologists and prevent unnecessary follow-up scans being performed.

The recent rapid advancement in the field of convolutional neural networks (CNNs) for image recognition has led to the possibility of applying these techniques to pulmonary nodule classification. Given the characteristic appearance of PFNs, their benign nature and their abundance in both screening and clinical settings, they are an interesting target for the training of a CNN classifier. Here, we evaluate the performance of a new deep convolutional neural network for PFN classification (PFN-CNN) trained on nodules retrospectively collected from two European centers, including validation on a holdout dataset.

## Materials and methods

### Population and data selection

The main data used for the current study were collected as part of the public-private grant provided by the European Institute of Innovation and Technology (EIT), grant agreement No. 17189. The use of retrospective data from two participating centers, Oxford University Hospitals (OUH) and University Medical Center Groningen (UMCG), was approved by local Ethics Committees. The need for consent from individuals was waived by local Ethics Committees, as the study was retrospective in nature, and at the point of data selection, all participant data were anonymized.

Inclusion criteria were as follows: CT scans of men and women, aged 18 years or above, with solid lung nodules 5–15 mm in maximum axial diameter, having ground truth of either histology, subsequent resolution, or stability in volumetric size after 1 year follow-up or in diameter after 2-year follow-up [12]. Exclusion criteria were as follows: CT scans with motion artifacts, slice thickness of more than 2.5 mm, more than 5 nodules reported on the scan, or malignancy in the last 5 years. Depending on the measurement tool available at the time of data collection, the maximum axial diameter of a nodule was either derived from semi-automatic volumetry or measured manually using a digital caliper tool. Measurements were rounded to the nearest millimeter. Although the inclusion criteria for nodule size were 5–15 mm, some of the nodules, after enrolment in the study, were measured to be smaller due to measurement variability and were not excluded. These include seven 4 mm and two 3 mm nodules in the training set, and five 4 mm nodules in the test set.

### Curation of PFN data

The data comprised both clinical and screening data, based on CT scanners from different manufacturers, and a wide variety of protocols, including the possible use of contrast agent. Data from each center were marked up by a radiologist on-site, and curation was performed centrally to ensure consistency between sites. A typical PFN was defined according to de Hoop et al as “a fissure-attached, homogeneous, solid nodule with smooth margin and an oval, lentiform or triangular shape.” An atypical PFN was defined as “a nodule that either met all features but was not attached to visible fissure or a fissure-attached nodule, convex on one side and rounded on the other.” Non-PFN was defined as “a nodule with a shape that did not appear to be influenced by the fissure.” [6]. Due to high rate of discrepancy in distinguishing atypical PFNs and the higher probability of misclassifying malignant nodules as atypical PFNs compared to typical PFNs, only typical PFNs were considered to belong to the positive training class, and everything else was considered a negative. A total of 1,668 nodules from 1,260 individuals were selected from the two participating medical centers for training, validation, and testing. The split ratio between training/cross-validation and testing (holdout dataset) was approximately eight to one; 1557 nodules from 1103 individuals were used for training and cross-validation, while 196 nodules from 158 individuals were used for testing.

### Training of the PFN classifier

The CNN-based PFN classifier was developed by Optellum Ltd. and was initialized from a lung cancer prediction model trained on around 16,000 NLST nodule images, for the task of distinguishing malignant from benign nodules based on analyzing a cuboidal volume of CT data centered on each nodule. This pretraining step helps the network to learn general features associated with the related task of lung cancer prediction that can be reused for PFN classification. Details on the development of the lung cancer prediction model have been previously described [13, 14]. The PFN classifier was then fine-tuned using the curated PFN data to produce a PFN score for each presented nodule using five-fold cross-validation. For binary classification of typical PFNs/non-PFNs, the binarization threshold for the PFN score was determined from the training/cross-validation data, based on the Youden index.

### Evaluation of the PFN classifier

The PFN-CNN was validated by comparing its performance to human readers using a reader study. The testing dataset consisted of 196 nodules (from 158 individuals) detected on standard or low-dose CT. Patients whose data were not included in the training and cross-validation set were included in the reader study test set.

For the reader study, cuboidal patches of voxels with benign nodules at the center were presented to the PFN classification model. For each nodule, the PFN classifier provided a PFN score ranging from 0 to 100. Based on the PFN score produced by the PFN-CNN and the binarization threshold of 52.1, the nodules were classified as either typical PFN or non-PFN. Two radiologists with 8 (reader A) and 10 (reader B) years of experience in chest radiology and a 4th year radiology resident (reader C) were asked to label each nodule as typical PFN, atypical PFN, or non-PFN. To match the AI training procedure, nodules that were rated by two or more readers as typical PFNs were considered as positive, whereas atypical PFNs and non-PFNs were considered as negative results and were combined into one group.

### Data analysis

The Kruskal-Wallis test was used to determine whether a continuous variable was normally distributed. Normally distributed data were presented as mean and standard deviation (SD), and non-normally distributed data were presented as median and interquartile range (IQR). Mann-Whitney *U* test was used to compare continuous variables between nodule types. Model performance was compared to the consensus of readers and evaluated using receiver operating characteristics (ROC). The evaluation of agreement was presented using a confusion matrix and Cohen’s kappa, and was interpreted as follows: value ≤ 0, no agreement; 0.01–0.20, none to slight; 0.21–0.40, fair; 0.41–0.60, moderate; 0.61–0.80, substantial; and 0.81–1.00, almost perfect agreement [15]. All statistical analysis was performed using SPSS v23 (IBM).

## Results

### Internal cross-validation

Table 1 gives an overview of patient and nodule details from both training and test datasets. In the training dataset, 427/1,557 (27.4%) nodules were labeled as typical PFNs, and 1,045/1,557 (67.1%) nodules were labeled as non-PFNs. The mean AUC of five-fold cross-validation on the internal dataset was 91.9% (95% CI 90.6–92.9). At an optimal operating point of 52.1, the PFN-CNN yielded 87.4% (95% CI 86.2–88.4) accuracy, 78.7% (95% CI 75.8–81.5) sensitivity, and 90.4% (95% CI 89.1–91.5) specificity.

**Table 1:** Details of nodules and associated clinical data

	Training dataset		Testing dataset	
	Typical PFNs, *n* (%)	Atypical PFNs and non-PFNs, *n* (%)	Typical PFNs, *n* (%)	Atypical PFNs and non-PFNs, *n* (%)
Patients—age				
18 ≤ x ≤ 50	19 (6.2)	51 (6.4)	1 (2.7)	2 (1.7)
50 < x ≤ 60	126 (41.0)	252 (31.7)	8 (21.6)	25 (20.7)
60 < x ≤ 70	102 (33.2)	296 (37.2)	14 (37.8)	42 (34.7)
70 < x ≤ 80	44 (14.3)	137 (17.2)	8 (21.6)	24 (19.8)
80 < x ≤ 90	15 (4.9)	57 (7.2)	3 (8.1)	10 (8.3)
90 < x ≤ 100	1 (0.3)	3 (0.4)	3 (8.1)	18 (14.9
Missing	0 (0.0)	0 (0.0)	1 (2.7)	2 (1.7)
Patients—sex				
Female	34 (11.1)	259 (32.5)	6 (16.2)	40 (33.1)
Male	273 (88.9)	537 (67.5)	31 (83.8)	81 (66.9)
Nodules—location				
Left lingula lobe	17 (4.0)	62 (5.5)	0 (0.0)	5 (3.3)
Left lower lobe	110 (25.8)	288 (25.5)	12 (26.7)	33 (21.9)
Left upper lobe	19 (4.4)	123 (10.9)	2 (4.4)	16 (10.6)
Right lower lobe	97 (22.7)	294 (26.0)	12 (26.7)	45 (29.8)
Right middle lobe	135 (31.6)	136 (12.0)	13 (28.9)	17 (11.3)
Right upper lobe	49 (11.5)	227 (20.1)	6 (13.3)	35 (23.2)
Nodules—size				
< 5	2 (0.5)	7 (0.6)	2 (4.4)	3 (2.0)
5 ≤ x ≤ 7	344 (80.6)	832 (73.6)	32 (71.1)	104 (68.9)
7 ≤ x < 10	71 (16.6)	174 (15.4)	10 (22.2)	32 (21.2)
10 ≤ x < 15	10 (2.3)	112 (9.9)	1 (2.2)	10 (6.6)
> 15	0 (0.0)	5 (0.4)	0 (0.0)	2 (1.3)

### Demographics of the testing dataset

In total, 196 unique nodules from 158 participants were included. Mean age was 66.6 ± 8.7 years, and 48/158 (30.4%) participants were female. Based on the reader consensus, 45 (22.9%) were typical PFNs, 42 (21.4%) atypical PFNs, and 105 (53.6%) non-PFNs. Four (2.0%) nodules had no classification due to disagreement (Table 2). Significant difference (*p* = 0.02) was found in maximum axial diameter between typical PFNs (median 6 mm (IQR 5–7)) and atypical PFN/non-PFNs (media 6 mm (IQR 6–8)).

**Table 2:** Nodule classification by readers

	Typical PFN, *N* (%)	Atypical PFN, *N* (%)	Non-PFN, *N* (%)
Reader A	49 (25.0)	31 (15.8)	116 (59.2)
Reader B	46 (23.5)	57 (29.1)	93 (47.4)
Reader C	35 (17.9)	57 (29.1)	104 (53.1)
Consensus*	45 (22.9)	42 (21.4)	105 (53.6)

PFN, perifissural nodule

*Agreement between two or more readers (4 nodules are without agreement)

### PFN-CNN classifier performance

**Fig. 1:**
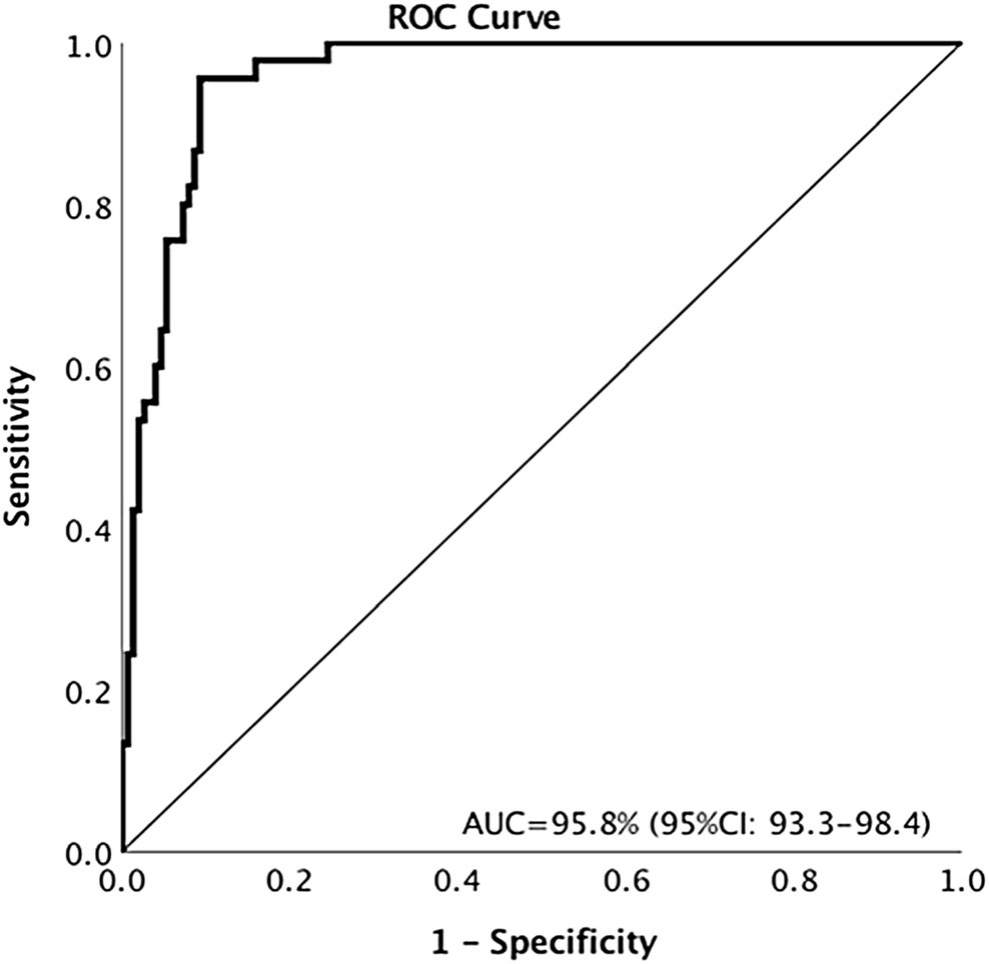
Performance of PFN-CNN for the classification of typical PFNs

**Fig. 2:**
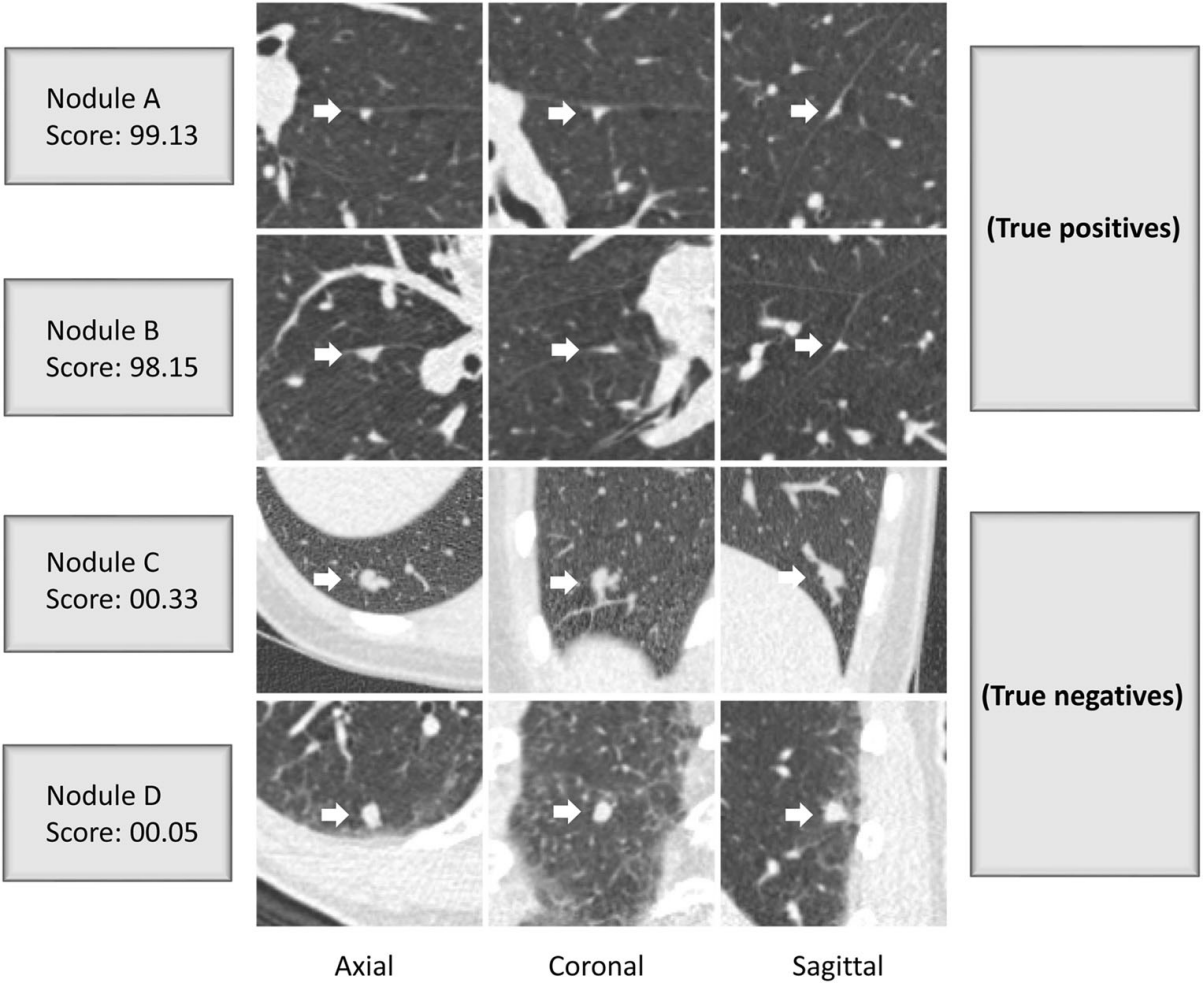
Examples from the test dataset along with scores generated by the PFN-CNN

**Fig. 3:**
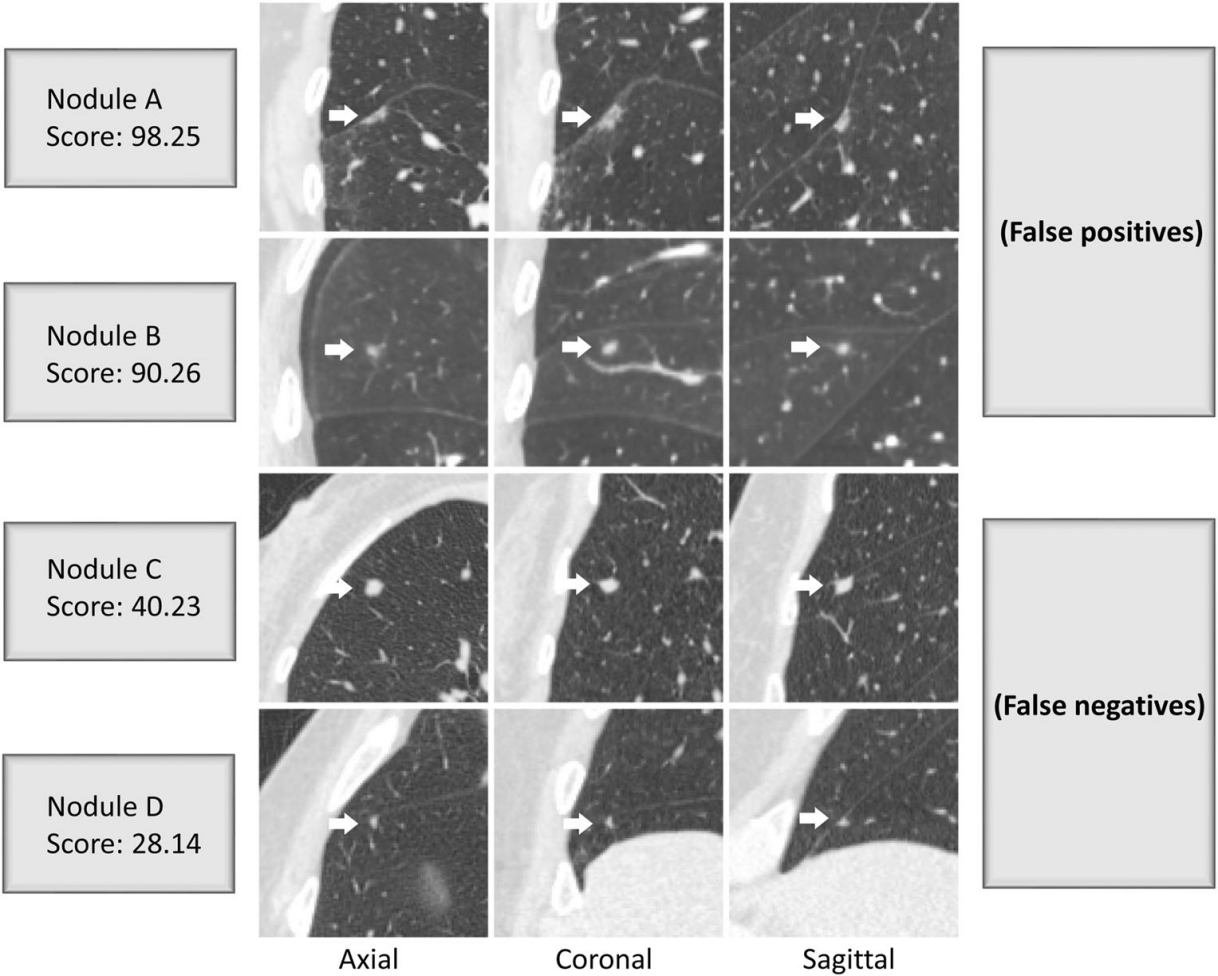
Examples from the test dataset along with scores generated by the PFN-CNN

When the performance in classifying typical PFNs was compared to the consensus of the three readers, the AUC of PFN-CNN was 95.8% (95% CI 93.3–98.4) (Fig. 1). The threshold value of 52.1 from the internal validation was used to distinguish typical PFNs from non-PFNs, which gave 89.8% accuracy, 95.5% (95% CI 84.9–99.5) sensitivity, and 88.1% (95% CI 81.8–92.8) specificity (Table 3). For the four pulmonary nodules without agreement in their classification as typical PFN, atypical PFN, or non-PFN, the PFN-CNN classified three as typical PFN and one as non-PFN (Figs. 2 and 3).

**Table 3:** Performance of readers and PFN-CNN compared to the reader consensus

	Sensitivity (%)	Specificity (%)	PPV (%)	NPV (%)
Reader A	93.3 (85.2, 100.0)	95.4 (91.9, 98.1)	85.7 (75.0, 94.9)	98.0 (95.3, 100.0)
Reader B	73.3 (59.1, 86.1)	98.7 (96.7, 100.0)	94.3 (85.7, 100.0)	92.5 (88.1, 96.2)
Reader C	95.6 (88.6, 100.0)	98.0 (95.5, 100.0)	93.5 (85.7, 100.0)	98.7 (96.5, 100.0)
PFN-CNN	95.6 (88.5, 100.0)	88.1 (83.0, 93.0)	70.5 (59.6, 81.4)	98.5 (96.1, 100.0)

*PFN-CNN*, convolutional neural network for the classification of perifissural nodules; *PPV*, positive predictive value; *NPV*, negative predictive value

### Reader agreement

The agreement was substantial between the reader consensus and PFN-CNN, with 89.8% (95% CI 85.7–93.9) accuracy and 0.744 (95% CI 0.635–0.842) kappa. There was substantial agreement between the PFN-CNN and the three individual readers (Table 4), and kappa (*k*) was 0.748 (95% CI 0.850–0.646), 0.623 (95% CI 0.745–0.501), and 0.732 (95% CI 0.838–0.626) for readers A, B, and C, respectively. Inter-reader agreement was substantial between readers: reader A and reader B, *k* = 0.639 (95% CI 0.510–0.768), reader A and reader C, *k* = 0.792 (95% CI 0.892–0.692), and reader B and C, *k* = 0.706 (95% CI 0.829–0.583).

**Table 4:** Confusion matrix and kappa on the agreement between the PFN-CNN and the three readers in typical PFN classification

		PFN-CNN			Kappa (95%CI)
		Typical PFN	Non-PFN	Total	
Reader A	Typical PFN	45	4	49	0.748 (0.850, 0.646)
	Non-PFN	16	131	147	
	Total	61	135	196	
Reader B	Typical PFN	34	1	35	0.623 (0.745, 0.501)
	Non-PFN	27	134	161	
	Total	61	135	196	
Reader C	Typical PFN	43	3	46	0.732 (0.838, 0.626)
	Non-PFN	18	132	150	
	Total	61	135	196	
Consensus	Typical PFN	43	2	45	0.744 (0.635, 0.842)
	Non-PFN	18	133	151	
	Total	61	135	196	

*PFN-CNN*, convolutional neural network for the classification of perifissural nodules

## Discussion

With this study, we have demonstrated the feasibility of a PFN-CNN to classify typical perifissural nodules on a chest CT. The PFN-CNN was trained on datasets from two European centers and built on top of a nodule characterization framework that had been trained on the US NLST data. The PFN-CNN showed excellent performance in identifying typical PFNs when compared to the consensus of three readers (AUC = 95.8%, sensitivity = 95.6%, specificity = 88.1%) and had comparable agreement with readers (classifier-reader agreement 85.7–89.8% [*k* = 0.62–0.75], inter-reader agreement 87.8–89.3% [*k* = 0.64–0.79]). This result demonstrates the potential utility of CNN-based systems for automatic PFN classification to reduce the workload of radiologists.

An interesting finding in our study was the significant improvement in performance in the reader study, with AUC = 95.8 (95% CI 93.3–98.4) as compared to the internal cross-validation, with AUC = 91.9% (95% CI 90.6–92.9). This improvement could have been caused by the difference in data mark-up available for classifying PFNs for training (binary labels) and for the reader study (tertiary labels). As both datasets contained a spectrum of cases, there may have been more borderline cases “rounded-up” or “rounded-down” for binary classification than in the tertiary classification.

Previous studies have shown that PFNs in both clinical and screening settings represent non-malignant lesions for which no follow-up is needed and that they account for 20–30% of (baseline) nodules [6–8]. Up to now, the gold standard for their identification has been the expert opinion of radiologists. However, there is an intrinsic variability in classification by radiologists, as has been shown by Schreuder et al who found only moderate agreement between radiologists [16]. Therefore, 100% accuracy may not be achievable. Automatic classification of PFNs using CNN has been previously attempted in only one study [17]. Ciompi et al used a CNN pre-trained using natural images to classify PFNs with respectable success (AUC = 86.8%). The difference in performance between their study and ours may be due to three factors. First, one of the major differences between our study and the work from Ciompi et al was the data used for pretraining; in our study, we used domain-specific images, whereas they used natural images for pretraining. Although we were not able to directly compare how different pretraining methods may influence the performance of neural network, we believe that the pretraining on images from the same domain on a related task may be a factor in the increased performance. Second, our PFN-CNN was not tested on an external dataset, which could potentially yield lower performance. Third, the definition of positive results was different between their study and ours. We focused on the identification of only typical PFNs and considered atypical PFNs as negative result. This is due to the higher disagreement of atypical PFNs among readers than typical PFNs [16], and the relatively low prevalence of atypical PFNs (3.1%) compared to typical PFNS (19.7%) [6].

Although our study demonstrates excellent performance in terms of AUC in classifying typical PFNs, there are several shortcomings. Firstly, the PPV of the described PFN-CNN was 70.5%. To our knowledge, our study is the first study to describe the PPV for an automatic PFN classifier. In a clinical setting, accurate identification of PFNs is critical to avoid accidental rule out of malignant nodules. In this case, 29.5% of nodules that were classified by the PFN-CNN as typical PFN were not typical PFNs. There are several reasons for the limited performance in PPV. Firstly, the binarization threshold of the PFN score was not optimized for PPV; rather, we used the Youden index to define the optimal cutoff for typical PFN classification, which equally values the performance in both sensitivity and specificity. Secondly, by using the consensus of three trained readers, the ground truth for typical PFN may be more conservative compared to the clinical practice, where usually a single reader makes the classification. This applies similarly to the data on which the PFN classifier was trained. For future studies, we suggest using the consensus of multiple readers for the curation of the CNN training data. Thirdly, the binary nature of the PFN-CNN and the grouping of atypical PFNs as negative results do not reflect the classification method suggested by de Hoop et al [6]. On the other hand, our study has demonstrated the importance of distinguishing the difference between typical and atypical PFNs, which has led to a better performance of the CNN compared to previous studies [17]. Use of the PFN-CNN, even though only focusing on typical PFNs, could potentially allow the exclusion of 22.9% of lung nodules in our dataset from further evaluation. When PFN-CNN is implemented as a part of a fully automated CAD system, the efficiency of lung cancer screening could be greatly increased. Lastly, the PFN-CNN was trained on the data provided by two participating centers. Whether the results can be generalized to other datasets from a different population and acquired with different CT scanners and protocols was not determined. However, as our training data was heterogeneous including both incidental and screening data, with a variety of CT scanners and protocols used, we do not expect drastic differences in performance when tested on an external dataset, though this has yet to be confirmed. Our immediate next step would be to focus on the automatic classification of atypical PFNs, as these are also more difficult for radiologists distinguish.

In conclusion, a PFN-CNN trained on datasets from European centers showed excellent performance in classifying typical PFNs in a holdout dataset in terms of AUC. This result demonstrates the potential utility of CNN-based systems in clinical and screening setting for ruling out benign pulmonary nodules and reducing radiologist workload.

## Supplementary Information

ESM 1 (DOCX 20 kb)

## Abbreviations

CNN: Convolutional neural network
GGO: Ground glass opacity
IQR: Interquartile range
NLST: National Lung Screening Trial
PFN: Perifissural nodule
ROC: Receiver operating characteristic
STD: Standard deviation
VDT: Volume doubling time
