# Supplementary material for: Evaluation of a novel deep learning–based classifier for perifissural nodules
Source: Eur Radiol. 2020 Dec 2;31(6):4023–30. doi: 10.1007/s00330-020-07509-x (PMC8128854; doi:10.1007/s00330-020-07509-x)
Supplement: Supplementary file 1 — (DOCX 20 kb) [file 330_2020_7509_MOESM1_ESM.docx]

Appendix

# Image parameters for training dataset

**Contrast**

| **Has contrast** | **Number of nodule images** |
| --- | --- |
| No | 2640 |
| Yes | 680 |

**Slice Thickness (**top 5 + Other**)**

| **Slice thickness (mm)** | **Number of nodule images** |
| --- | --- |
| 1 | 1768 |
| 0.625 | 995 |
| 2.5 | 374 |
| 1.25 | 130 |
| 2 | 23 |
| Other | 30 |

**Convolution Kernels (**top 5 + Other**)**

| Convolution kernel | Number of nodule images |
| --- | --- |
| B30f | 1670 |
| CHST | 479 |
| SOFT | 341 |
| BONE | 235 |
| STANDARD | 197 |
| Other | 398 |

**Scanners** (Showing top 3 plus other per manufacturer and excluding manufacturers with less than 3 nodule images)

| **Manufacturer** | **Models** | **Number of Nodule Images** |
| --- | --- | --- |
| GE Medical Systems | LightSpeed VCT | 629 |
|  | Revolution HD | 320 |
|  | Revolution GSI | 122 |
|  | Other | 428 |
|  | **Total** | 1499 |
| Siemens | Sensation 64 | 957 |
|  | Sensation 16 | 689 |
|  | Definition | 22 |
|  | Other | 44 |
|  | **Total** | 1712 |
| Toshiba | Aquilion | 95 |
|  | Aquilion ONE | 11 |
|  | **Total** | 106 |

# Image parameters for reader study dataset

**Contrast**

| Has contrast | Number of nodule images |
| --- | --- |
| No | 157 |
| Yes | 39 |

**Slice Thickness**

| **Slice thickness (mm)** | **Number of nodule images** |
| --- | --- |
| 1 | 104 |
| 0.625 | 61 |
| 1.25 | 21 |
| 2 | 10 |

**Convolution Kernels** (top 5 + Other)

| Convolution kernel | Number of nodule images |
| --- | --- |
| B30f | 98 |
| CHST | 47 |
| SOFT | 25 |
| BONEPLUS | 10 |
| STANDARD | 6 |
| Other | 10 |

**Scanners** (top 3 + Other, per manufacturer)

| **Manufacturer** | **Models** | **Number of Nodule Images** |
| --- | --- | --- |
| GE Medical Systems | LightSpeed VCT | 71 |
|  | LightSpeed Pro 16 | 10 |
|  | Revolution GSI | 7 |
|  | Other | 4 |
|  | **Total** | 92 |
| Siemens | Sensation 64 | 97 |
|  | Sensation 16 | 2 |
|  | SOMATON Definition Flash | 1 |
|  | **Total** | 100 |
| Toshiba | Aquilion | 3 |
|  | Aquilion ONE | 1 |
|  | **Total** | 4 |
